# The role of the open abdomen procedure in managing severe abdominal sepsis: WSES position paper

**DOI:** 10.1186/s13017-015-0032-7

**Published:** 2015-08-12

**Authors:** Massimo Sartelli, Fikri M. Abu-Zidan, Luca Ansaloni, Miklosh Bala, Marcelo A. Beltrán, Walter L. Biffl, Fausto Catena, Osvaldo Chiara, Federico Coccolini, Raul Coimbra, Zaza Demetrashvili, Demetrios Demetriades, Jose J. Diaz, Salomone Di Saverio, Gustavo P. Fraga, Wagih Ghnnam, Ewen A. Griffiths, Sanjay Gupta, Andreas Hecker, Aleksandar Karamarkovic, Victor Y. Kong, Reinhold Kafka-Ritsch, Yoram Kluger, Rifat Latifi, Ari Leppaniemi, Jae Gil Lee, Michael McFarlane, Sanjay Marwah, Frederick A. Moore, Carlos A. Ordonez, Gerson Alves Pereira, Haralds Plaudis, Vishal G. Shelat, Jan Ulrych, Sanoop K. Zachariah, Martin D. Zielinski, Maria Paula Garcia, Ernest E. Moore

**Affiliations:** Department of Surgery, Macerata Hospital, Via Santa Lucia 2, 62100 Macerata, Italy; Department of Surgery, College of Medicine and Health Sciences, UAE University, Al-Ain, United Arab Emirates; General Surgery I, Papa Giovanni XXIII Hospital, Bergamo, Italy; Trauma and Acute Care Surgery Unit, Hadassah Hebrew University Medical Center, Jerusalem, Israel; Department of Surgery, Hospital San Juan de Dios, La Serena, Chile; Department of Surgery, University of Colorado, Denver Health Medical Center, Denver, USA; Emergency Surgery Department, Maggiore Parma Hospital, Parma, Italy; Emergency Department, Niguarda Ca’ Granda Hospital, Milan, Italy; Division of Trauma, Surgical Critical Care, Burns, and Acute Care Surgery, University of California San Diego Health Science, San Diego, USA; Department of Surgery, Tbilisi State Medical University, Kipshidze Central University Hospital, Tbilisi, Georgia; Trauma, Emergency Surgery, Surgical Critical Care, University of Southern California, Los Angeles, USA; Shock Trauma Center, University of Maryland School of Medicine, Baltimore, USA; Trauma Surgery Unit, Maggiore Hospital, Bologna, Italy; Division of Trauma Surgery, Hospital de Clinicas, School of Medical Sciences, University of Campinas, Campinas, Brazil; Department of Surgery Mansoura, Faculty of Medicine, Mansoura University, Mansoura, Egypt; Department of Surgery, Queen Elizabeth Hospital, Birmingham, UK; Department of Surgery Government Medical College and Hospital, Chandigarh, India; Department of General and Thoracic Surgery, University Hospital of Giessen, Giessen, Germany; Clinic for Emergency Surgery, Faculty of Medicine, University of Belgrade, Belgrade, Serbia; Department of Surgery, Edendale Hospital, Pietermaritzburg, Republic of South Africa; Department of Visceral, Thorax and Transplant Surgery, University of Innsbruck, Innsbruck, Austria; Department of General Surgery, Rambam Health Care Campus, Haifa, Israel; Department of Surgery, Trauma Research Institute, University of Arizona, Tucson, AZ USA; Abdominal Center, University Hospital Meilahti, Helsinki, Finland; Department of Surgery, Yonsei University College of Medicine, Seoul, South Korea; Department of Surgery, University Hospital of the West Indies, Kingston, Jamaica; Department of Surgery, Post-Graduate Institute of Medical Sciences, Rohtak, India; Department of Surgery, University of Florida, Gainesville, FL USA; Department of Surgery, Fundación Valle del Lili, Hospital Universitario del Valle, Universidad del Valle, Cali, Colombia; Division of Emergency and Trauma Surgery, Ribeirão Preto Medical School, Ribeirão Preto, Brazil; Department of General and Emergency Surgery, Riga East Clinical University Hospital “Gailezers”, Riga, Latvia; Department of Surgery, Tan Tock Seng Hospital, Singapore, Singapore; 1st Surgical Department of First Faculty of Medicine, General University Hospital, Prague Charles University, Prague, Czech Republic; Department of Surgery, MOSC Medical College Kolenchery, Cochin, India; Department of Surgery, Mayo Clinic, Rochester, MN USA; Centro de investigaciones clínicas, Fundación Valle del Lili, Cali, Colombia

## Abstract

The open abdomen (OA) procedure is a significant surgical advance, as part of damage control techniques in severe abdominal trauma. Its application can be adapted to the advantage of patients with severe abdominal sepsis, however its precise role in these patients is still not clear.

In severe abdominal sepsis the OA may allow early identification and draining of any residual infection, control any persistent source of infection, and remove more effectively infected or cytokine-loaded peritoneal fluid, preventing abdominal compartment syndrome and deferring definitive intervention and anastomosis until the patient is appropriately resuscitated and hemodynamically stable and thus better able to heal.

However, the OA may require multiple returns to the operating room and may be associated with significant complications, including enteroatmospheric fistulas, loss of abdominal wall domain and large hernias.

Surgeons should be aware of the pathophysiology of severe intra-abdominal sepsis and always keep in mind the option of using open abdomen to be able to use it in the right patient at the right time.

## Introduction

The open abdomen (OA) procedure is one of the greatest surgical advances in recent times and may have enormous application in the daily management of critically ill surgical patients. The OA may be a useful option for treating patients with abdominal sepsis. However its precise role in these patients is still not clear.

On the basis of the source and nature of the microbial contamination, peritonitis can be classified into primary, secondary and tertiary [1].

*Primary peritonitis* is a diffuse bacterial infection without loss of integrity of the gastrointestinal tract. It is a rare condition occurring mainly in infancy, early childhood and in cirrhotic patients.

*Secondary peritonitis* is the most common form of peritonitis and results from loss of integrity of the gastrointestinal tract due to perforation (e.g. perforated duodenal ulcer) or by direct invasion from infected intra-abdominal viscera (e.g. gangrenous appendicitis) [2].

*Tertiary peritonitis* is defined as a severe recurrent or persistent intra-abdominal infection >48 h after apparently successful and adequate surgical source control of secondary peritonitis [3]. Although it is less common, it may comprise of a severe systemic inflammation response [4]. Tertiary peritonitis is associated with microbial shift towards nosocomial flora including *Staphylococci coagulase*-*negative*, *Candida*, *Enterococci*, *Pseudomonas*, *Enterobacter* and other opportunistic bacteria and fungi [3, 4]. Mortality rate in tertiary peritonitis is very high, ranging from 30 to 64 % [4].

Abdominal sepsis is the host’s systemic inflammatory response to bacterial or yeast peritonitis [5].

Sepsis from an abdominal origin is initiated by the outer membrane component of gram-negative organisms (e.g., lipopolysaccharide [LPS], lipid A, endotoxin) or gram-positive organisms (e.g., lipoteichoic acid, peptidoglycan), as well toxins from anaerobic bacteria [6]. This leads to the release of proinflammatory cytokines such as tumor necrosis factor α (TNF-α), and interleukins 1 and 6 (IL-1, IL-6). TNF-α and interleukins lead to the production of toxic mediators [6], which may cause a complex, multifactorial syndrome that may evolve into conditions of varying severity and may lead to the functional impairment of one or more vital organs or systems [7].

Severe sepsis is defined as sepsis associated with organ dysfunction or tissue hypoperfusion [8]. Whereas, septic shock is defined as severe sepsis associated with refractory hypotension despite adequate fluid resuscitation [8]. Previous studies have demonstrated that mortality rates increase dramatically in patients with severe sepsis and septic shock [9] and that aggressive treatment of these patients may improve outcomes [10]. Mortality rates have recently stabilized due to advances in treatment options that manage the underlying infection and supports failing organs, however they remain high [11].

In 2014 the definitive data from the CIAOW study (Complicated intra-abdominal infections worldwide observational study) were published [12]. The study describes the epidemiological, clinical, and treatment profiles of complicated intra-abdominal infections in a worldwide context. The overall mortality rate was 10.5 %. Analyzing the subgroups of patients with severe sepsis and septic shock at hospital admission the mortality rate reached 36.5 %.

There are many risk factors that most commonly may precipitate severe sepsis and septic shock, including advanced age, acquired immunodeficiency syndrome, and use of immunosuppressive agents [11]. However, organ failure risk factors generally include the causative organism and the patient’s genetic composition, underlying health status, and pre-existing organ function [11].

It has been observed that in some patients, peritonitis may trigger an excessive immune response and sepsis may quickly and progressively evolve into severe sepsis, septic shock, and finally to multi organ failure [13].

These patients may benefit from aggressive surgical treatment and source control following an initial emergency laparotomy to control the local inflammatory response.

Three strategies in the management of these difficult patients have been reported [2, 14]:

- Relaparotomy on demand (when required by the patient’s clinical condition)
- Planned relaparotomy in the 36-48-h post-operative period (when relaparotomy is planned after first operation)
- Open abdomen procedure

Choosing the best option is not a simple task. In 2007, van Ruler et al., published a randomized clinical trial comparing on-demand vs. planned relaparotomy strategy in patients with severe peritonitis. Patients in the on-demand relaparotomy group did not have a significantly lower mortality rate or major peritonitis-related morbidity compared with the planned relaparotomy group but they had a substantial reduction in re-laparotomies, health care utilization, and medical costs [15]. However accurate and timely identification of patients who need a relaparotomy is a very difficult decision-making process. At present there are no clinical criteria to select patients for a relaparotomy [14].

Open abdomen procedure (OA), is defined as intentionally leaving the fascial edges of the abdomen un-approximated (laparostomy). The abdominal contents are exposed and protected with a temporary coverage [16]. The OA technique, when used appropriately, may be useful in the management of surgical patients with severe abdominal sepsis (severe sepsis/septic shock) [17]. However, the role of the OA in the management of severe peritonitis is still being debated [18].

Robledo et al. compared open versus closed abdomen procedures in 40 patients with severe secondary peritonitis [18]. They found no significant difference in mortality rates (55 % open versus 30 % closed), nevertheless, the relative risk and odds ratio for death in the open group (1.83 and 2.85, respectively) led to termination of the study at the first interim analysis. However, in that study, the temporary abdominal closure was accomplished by a sandwich technique with non-absorbable mesh sutured to the fascia.

Although current clinical guidelines suggest that OA technique should not be used routinely, but individualized for each patient with abdominal sepsis [19], OA may be an important option in surgical management of severe peritonitis [20].

The management of the OA can be safely achieved with acceptable outcomes but it remains expensive [21]. Its overuse may potentially lead to increased morbidity, of which enteroatmospheric fistulas are the most serious complication [22]. Compared with trauma patients, patients with abdominal sepsis have been described to have worse outcomes after OA, with increased incidence of fistula formation, intra abdominal abscesses, and a higher delayed primary closure rate [12]. A correct management of the OA procedure is crucial to reduce the associated complications.

Due to patients heterogeneity included within individual studies, an OA classification system was proposed by Bjorck et al. in 2009 [23] and updated by Kirkpatrick et al. [19] (Appendix 1).

## Decision making process for leaving the abdomen open in patients with abdominal sepsis

Outcomes of patients with severe sepsis/septic shock due to abdominal source are related to early aggressive hemodynamic support, prompt surgical source control and early and adequate antimicrobial therapy.

Sepsis source control is based on three principles: drainage and lavage of the infected fluid or other collections, debridement of infected/necrotic tissue and definitive or temporary measures to correct anatomic derangements (for example closure of perforated viscus) and to restore optimal function [24]. In critically ill patients with severe sepsis these principles can be applied at different times in the same patient.

Deciding whether to complete the initial operation or perform an abbreviated surgery in critically ill patients is an important and complex decision. The OA concept is closely linked to damage control surgery, and may be easily adapted to patients with advanced sepsis and can incorporate the principles of the Surviving Sepsis Campaign [8]. In these patients an OA approach may be required for different reasons including: controlling any persistent source of infection, preventing abdominal compartment syndrome and deferring definitive intervention and anastomosis.

### Damage control surgery in patients with severe sepsis

The origins of damage control surgery was initially developed in the 1980s by Stone at the Grady General Hospital (Atlanta, GA, USA) [25], and detailed by Burch at the Ben Taub General Hospital (Houston, TX, USA) in the early 1990s [26]. The abbreviated laparotomy for trauma patients was defined as the initial control of surgical bleeding by simple operative techniques such as packing etc. for life saving techniques. The patient was taken to ICU where subsequent resuscitation corrected hypothermia, acidosis, and coagulopathy. Once the patient had regained their physiologic reserve, definitive re-exploration and reconstructive surgery was performed with or without final abdominal closure. This type of management can be successfully applied for severe abdominal sepsis including OA technique.

Patients may progress to severe sepsis and septic shock having progressive organ dysfunction, hypotension, myocardial depression and then coagulopathy. These patients are hemodynamically unstable and clearly not optimal candidates for immediate complex operative interventions [27]. After initial surgery, the patient is rapidly taken to the ICU for physiologic optimization. Early treatment with aggressive hemodynamic support can limit the damage of sepsis-induced tissue hypoxia and may limit the over stimulation of endothelial activity [8]. Following the early hemodynamic support, in principle after 24–48 h, reoperation may be performed with or without final abdominal closure.

### Relaparotomy and ‘relook’

OA facilitates repeated abdominal exploration in the patients with peritonitis and severe sepsis/septic shock. Reoperations, in managing patients with severe sepsis/septic shock due to severe peritonitis, are common and may be useful in attenuating the inflammatory response of patients with ongoing infections.

In some patients, peritoneal infection may quickly lead to an excessive inflammatory response, causing organ failure. In these patients, an early reintervention with surgical lavage of the peritoneal cavity and evacuation of toxic content and inflammatory cytokines may be crucial for stopping the septic cascade. This allows better control of the local inflammatory response and improved outcomes.

Several studies have evaluated clinical variables that may be associated with the need for relaparotomy in the immediate post-operative period [28–31]. In a retrospective study of 219 consecutive patients who underwent emergency laparotomy for secondary peritonitis, van Ruler et al. [28] showed that both the origin of secondary peritonitis and findings at emergency laparotomy were poor indicators for an early relaparotomy. Signs of progressive or persistent organ failure during early postoperative period were the best indicators for ongoing infection.

In a retrospective study involving 523 consecutive patients with secondary peritonitis, Koperna et al. [29] evaluated outcomes of 105 patients in whom standard surgical treatment of secondary peritonitis failed and who had to undergo relaparotomy for persisting abdominal sepsis (study group). While there were no differences in mortality between “planned relaparotomy” and “relaparotomy on demand”, re-exploration performed more than 48 h after the initial operation resulted in a significantly higher mortality rate (76.5 % versus 28 %; p = 0.0001). The lowest mortality rate (9 %) was achieved in patients who underwent reoperation within 48 h. The results of this study showed that timely relaparotomy should be done early and within 48 h.

The final decision to perform a re-operation on a patient in the on-demand setting is complex and generally it is based on the patient generalized septic response and on the lack of clinical improvement during early postoperative period [5, 28]. The on-demand strategy implies a vigilant observation of the patient and includes reoperation when patients show clinical deterioration or do not improve [32]. However, these conditions are not well defined [33] and often relaparotomy may be performed too late. In patients with severe sepsis and septic shock, OA allows easy second-look to control the source of infection and evacuate inflamed and toxic content, reducing the load of peritoneal cytokines and other inflammatory substances and preventing their production by removing the source itself.

### Prevention of abdominal compartment syndrome

The systemic inflammatory response syndrome, increased vascular permeability and aggressive crystalloid resuscitation predispose to fluid sequestration with formation of peritoneal fluid. Patients with advanced sepsis commonly develop shock bowel resulting in excessive bowel oedema. These changes and associated forced closure of the abdominal wall may result in increased intra-abdominal pressure (IAP) ultimately leading to intra-abdominal hypertension (IAH) [34]. Although recognized over 150 years ago, the pathophysiologic implications of elevated IAP have essentially been rediscovered only within the last two decades [35, 36]. In 2013, the World Society of the Abdominal Compartment Syndrome has published practice guidelines for the management of intra-abdominal hypertension [19]. IAP was classified into four grades:

- Grade I IAP 12–15 mmHg
- Grade II IAP 16–20 mmHg
- Grade III IAP 21–25 mmHg
- Grade IV IAP > 25 mmHg

Elevated IAP commonly causes marked deficits in both regional and global perfusion that may result in significant organ failure. An uncontrolled IAH, with an IAP exceeding 20 mmHg and a new organ failure onset leads to abdominal compartment syndrome (ACS) [19]. This in turn has further effects on intra-abdominal organs, as well as indirect effects on remote organ(s) and system(s). ACS is a potentially lethal complication characterized by effects on splanchnic, cardiovascular, pulmonary, renal, and central nervous systems [19]. Ventricular filling is reduced as a result of decreased venous return caused by the compression of the inferior vena cava or portal vein. Preload measurements such as central venous pressure (CVP) and pulmonary artery occlusion pressure (PAOP) may be falsely elevated. Critical clinical conditions play an important role in aggravating the effects of elevated IAP and may reduce the threshold of IAH that causes the clinical manifestations of ACS. The combination of IAH and the physiological effects of severe sepsis and septic shock may result in high morbidity and mortality rates [16]. Especially in the case of severe peritonitis the physiological effect of ACS to gastrointestinal tract may aggravate the abdominal sepsis. Specifically the mucosal-barrier function is altered causing increased permeability and bacterial translocation [37].

The earliest and potentially most effective means of treating ACS in high-risk patient is its recognition and prompt intervention. Presumptive decompression should be considered at the time of laparotomy in patients who demonstrate risk factors for IAH/ACS [37]. The decision to perform a laparostomy in patients with abdominal sepsis is usually based on the intraoperative judgment of the surgeon without IAP measurements during the operation.

### Delayed intestinal anastomosis

An additional advantage of the OA strategy in abdominal sepsis is to delay the bowel anastomosis [38] and the potential to avoid stoma formation.

The staged laparotomy was initially described in trauma setting with resection of injured bowel without anastomosis and returning to complete GI reconstruction once the patient is stable [39].

In patients with severe secondary peritonitis and significant hemodynamic instability and compromised tissue perfusion, primary anastomosis is at high risk for anastomotic leakage resulting in increased mortality. In these patients, consideration should be given to initially control the source of peritoneal contamination and delay the bowel anastomosis. In a retrospective study by Ordonez et al., 112 patients with secondary peritonitis requiring bowel resection who were managed with staged laparotomy were analyzed [40]. Deferred primary anastomosis was performed in 34 patients where the bowel ends were closed at first operation. Definitive anastomosis were reconstructed at the subsequent operation after physiological stabilization in the intensive care unit (ICU) and repeated peritoneal washes until the septic source was controlled. In contrast, 78 patients underwent small bowel or colonic diversion followed by similar ICU stabilization and peritoneal washes. In both groups, the abdomens were left open at the initial operation and a Velcro system or vacuum pack was used for temporary abdominal closure. The mean number of laparotomies was four in both groups. There were more patients with colon resections in the diversion group (80 % vs. 47 %). There was no significant difference in hospital mortality (12 % for deferred anastomosis vs. 17 % for diversion), frequency of anastomotic leaks or fistulas (9 % vs. 5 %), or ARDS (18 % vs. 31 %). The authors concluded that in critically ill patients with severe secondary peritonitis managed with staged laparotomies, deferred primary anastomosis can be performed safely as long as adequate control of the septic foci and restoration of deranged physiology is achieved prior to reconstruction.

A high rate of intestinal reconstruction and a low mortality rate was demonstrated in a prospective study by Kafka et al. [41] in patients with generalized peritonitis (Hinchey III and IV) treated with initial damage control surgery and vacuum assisted abdominal closure.

Among 51 patients, bowel continuity was restored in 38 patients, in four protected by a loop ileostomy. Five anastomotic leaks (13 %) were encountered requiring loop ileostomy (two patients) or Hartmann’s procedure (three patients). Postoperative abscesses were seen in four patients, abdominal wall dehiscence in one and re-laparotomy for drain-related small bowel perforation in one. The overall mortality rate was 10 %. 35/46 (76 %) of the surviving patients left the hospital with reconstructed colon continuity. Fascial closure was achieved in all patients.

In patients with advanced sepsis, open abdomen allows surgeons to abbreviate initial surgery in the face of severe physiological compromise, relook surgery in patients with ongoing sepsis, preventing abdominal compartment syndrome and delay intestinal anastomosis until the patient is appropriately resuscitated and hemodynamically stable. OA may be a useful surgical option in those patients having severe sepsis and septic shock. An “on demand” strategy may be reserved for more stable patients.

## Management of patients with open abdomen

### Source control

The first stage of open abdomen procedure in managing abdominal sepsis is an adequate and prompt source control. The primary objectives of surgical intervention include:

- determining the cause of peritonitis;
- draining fluid collections;
- controlling the origin of the abdominal sepsis.

Once an OA strategy is decided, the optimal method chosen for laparostomy should allow an easy re-entry to the abdominal cavity, and allow for expansion in order to prevent ACS.

### Resuscitation and antimicrobial therapy

The second stage of open abdomen procedures involves resuscitation, which should include fluid administration, vasopressive agents and adequate antimicrobial therapy.

Aggressive hemodynamic support can limit sepsis-induced tissue damage and prevent the over stimulation of endothelial activity. Current Surviving Sepsis guidelines emphasize the importance of traditional mean arterial pressure (MAP) >65 mmHg, central venous pressure (CVP) of 8–12 mmHg in combination with a central venous oxygen saturation (ScvO2) >70 % and Urine output >0.5 mL/kg/h [8].

Empiric antimicrobial therapy should be started as soon as possible in patients with severe sepsis with or without septic shock [8]. An inadequate antimicrobial regimen is associated with unfavorable outcomes in critical ill patients [42]. For these patients, a de-escalated approach may be the most appropriate strategy. A broad-spectrum antimicrobial regimen should be started in the initial stages of treatment. Subsequent modification (de-escalation) of the initial regimen becomes possible later, when culture results are available and clinical status can be better assessed. This is usually achieved 48–72 h after initiation of empiric therapy [42].

Antimicrobial therapy should be reassessed daily, because the pathophysiological changes may significantly affect antibiotics pharmacokinetics in the critically ill patients [42].

In these patients *Candida* species infection may be clinically significant and is usually associated with poor prognosis. Although appropriate antifungal therapy is essential for controlling invasive *Candida* infections and improving outcome, early diagnosis of invasive candidiasis remains a challenge because criteria for starting empirical antifungal therapy in critically ill patients are poorly defined [43].

Antifungal empirical treatment depends on the identification of patients at increased risk for invasive candidiasis such as patients with *candida* colonization and on the positive predictive value of risk assessment strategies, such as the colonization index, candida score, and predictive rules based on combinations of risk factors [44].

### Returning to the operating room

In principle, following 24 to 48 h after the initial surgery the patient should be taken back to the operating room for re-operation. Re-operation should be performed within 24–48 h after the initial surgery because exploration of peritoneal cavity with lavage, drainage and source control is feasible. The abdominal exploration may be more difficult later due to the intraperitoneal adhesions and risks enteric injury.

### Fascial closure

Following re-exploration, the goal is early and definitive closure of the abdomen, in order to reduce the complications associated with an open abdomen [45], such as enteroatmospheric fistulas, fascial retraction with loss of abdominal wall domain, and development of massive incisional hernias.

Early definitive closure is the basis of preventing or reducing the risk of these complications [45, 46].

The literature suggests a bimodal distribution of primary closure rates, with early closure depending on post-operative intensive care management and delayed closure depending on the choice of the temporary abdominal closure technique [47].

A systematic review and meta-analysis to evaluate whether early fascial abdominal closure had advantages over delayed approach was published in 2014 [48]. Mortality, complications, and length of stay were compared between early and delayed fascial closure. In total, 3125 patients were included for the final analysis, and 1942 (62 %) patients successfully achieved early fascial closure. Compared with delayed abdominal closure, early fascial closure was associated with reduced mortality (12.3 % versus 24.8 %, RR, 0.53, P < 0.0001) and complication rate (RR, 0.68, P < 0.0001). The study confirmed the clinical advantages of early fascial closure compared with delayed closure in treatment of patients with open abdomen.

Early fascial closure is commonly performed within 4–7 days days of the initial laparostomy [40]. Primary fascial closure can be achieved in many cases within few days from the initial operation without technical difficulties. Patients having abdominal sepsis are less likely to achieve an early fascial closure [49] it should be performed as soon as possible after severe abdominal sepsis is controlled [50].

Ideally, the fascia should be closed in patients whose adequate source control is performed with no further planned surgical intervention, severe sepsis is controlled and fascial closure is feasible without relevant increase of IAP.

However, patients undergoing primary fascial closure need strict monitor of their status to assess the possibility of a definitive operation. An early fascia-to-fascia closure can not be successful if early surgical source control fails [51].

Restrictive fluid resuscitation may be suggested for the hemodynamic support of critically ill patients with abdominal sepsis [5]. Some evidence exists to support implementing restrictive fluid therapy protocols in critically ill patients with abdominal sepsis and IAH [52].

In these patients, fluid resuscitation should be controlled to avoid fluids overload, which may aggravate gut oedema and lead to increased intra-abdominal pressure [5]. Reduction of bowel oedema with a conservative fluid resuscitation may increase the chances for early definitive abdominal closure. Intra-vesical measurements of IAP should be frequently performed in patients with severe sepsis or septic shock of abdominal origin, to monitor the trend of IAP and decide the timing of fascial closure.

It has been proposed that leaving patients with open abdomen on continuous neuromuscular blockade (NMB) can improve closure rates. However, the current evidence comparing continuous NMB to simple sedation is equivocal [53, 54]. Active diuresis is often used to decrease bowel and abdominal wall oedema and facilitate fascial closure. Yet, there is no convincing data to suggest that use of diuretics improves the rate or time to closure [19].

Delayed fascial closure is defined as fascial abdominal closure achieved 7 or more days after the initial OA procedure [47]. The delayed abdominal wall closure may be often problematic because of fascial edges lateralization leading to unfavourably high tensile midline forces. Abdominal wall closure should be performed by approximating the fascial edges progressively and incrementally, each time the patient undergoes surgery until it is completely closed.

### Temporary abdominal closure

The ideal temporary abdominal closure (TAC) method should protect the abdominal contents, prevent evisceration, allow removal of infected or toxic fluid from the peritoneal cavity, prevent the formation of fistulas, avoid damage to the fascia, preserve the abdominal wall domain, make re-operation easy, safe and facilitate definitive closure [55].

Many different techniques of TAC have been introduced during the past 10 years [56]. Numerous reports exist on all these techniques, but patient groups remain small, with a high heterogeneity, making comparison of techniques and outcomes difficult [56]. Various advantages and disadvantages of different forms of TAC are shown in Table 1. Although numerous TAC techniques have been applied in the setting of abdominal sepsis, many of those modalities are not primarily intended to close the infected abdomen, (e.g. skin only, meshes, or zipper)

**Table 1:** Advantages and disadvantages of different types of temporary abdominal closure (TAC) techniques

Technique	Equipment	Advantages	Disadvantages
Skin only closure	Skin staples, towel clips or sutures	Cheap, available, minimises heat and fluid loss	Damage to the skin, risk of evisceration, no control of fluid loss, incidence of ACS
‘Bogota’ bag	Sterile 3 litre Saline bag cut and shaped and sutured to fascial edges	Cheap, available, minimises heat and fluid loss	Damage to the fascial edges, risk of evisceration, no control of fluid loss. Allows some assessment of intestinal viability.
Opsite Sandwich technique	Polyethylene sheet, Opsite dressings, abdominal packs, 2 suction drains and wall suction.	Cheap, available, minimises heat and fluid loss is controlled and measurable	Incomplete fluid control and need for available wall suction.
Absorb_able_ mesh	Vicryl or similar MESH	Absorbable mesh, infection resistance, protects from evisceration, can be skin grafted.	High rate of subsequent incisional herniation
Non-absorbable mesh or commercial ‘Zipper’	Commerical Whittman patch	Abdominal re-exploration is easy, maintains abdominal domain, gradual abdominal closure possible	Commercial equipment required and multiple trips to the operating theatre usually required for closure.
Vacuum Assisted Closure (VAC)	Commercial equipment	Prevents loss of abdominal domain, collects and monitors fluid loss, decreases ACS, no damage to skin or abdominal fascia.	Expensive commercial equipment required. Usually requires GA to change VAC system

The first and easiest method to perform a laparostomy was the application of a plastic silo (the ‘Bogota bag’). This system is inexpensive, readily available and preserves the intact fascia when sutured to the skin edges. However, it does not provide sufficient traction to the wound edges and allows the fascial edges to retract laterally, resulting in difficult fascial closure under significant tension, especially if the closure is delayed [16].

### Negative pressure therapy (NPT)

Negative pressure therapy techniques have become the most extensively used methods for temporary abdominal wall closure.

It is an easy method and uses a fenestrated polyethylene sheet between the abdominal viscera and parietal peritoneum, followed by a moist towel, kerlix gauze bandage rolls with closed suction drains or a sponge covered with an occlusive adhesive drape and is known as the “vacuum pack technique” or the opsite sandwich technique [57]. This method is inexpensive, easily applied and changed, protects the viscera, prevents adhesions, removes exudate and prevents some loss of domain [58, 59]. Commercially prepared negative pressure dressings are available and the initial dressing may be changed to commercial dressing, if early closure is impossible.

NPT actively drains toxin or bacteria-rich intra-peritoneal fluid and has resulted in a high rate of fascial and abdominal wall closure [60]. Animal studies suggested that OA techniques using constant negative pressure to the peritoneal cavity may remove inflammatory ascites, reduce the systemic inflammatory response and improve organ injury [61]. NPT is still associated with high morbidity and high incidence of ventral hernia formation in surviving patients. This is caused by difficulties in definitive closure of the abdominal wall, especially after prolonged application of NPT. However, it is a highly promising method of temporary abdominal closure in the management of critically ill patients with abdominal sepsis [62, 63].

Enteroatmospheric fistulas formation is one of the most devastating complications of OA procedure and NPT. The development of fistulae correlates to duration of NPT and frequency of changing the dressing [64].

Nevertheless, recently published articles indicate that NPT can be applied for the successful treatment of enteroatmospheric fistulas [65] provided an adequate isolation and external drainage of the EAF is obtained. The rationale of obtaining an effective control and diversion of the fistula output is essential for a clean granulation of the exposed bowel and epithelization of the abdomen [66].

A practical algorithm for EAF management based on size, location, output and number of EAF’s (single or multiple), has been recently proposed [67].

The combination of NPT with fascial-approximation techniques using dynamic closure procedures to approximate fascial edges is safe and can facilitate delayed closure of open abdomen in septic patients [68, 69].

A systematic review and meta-analysis of the open abdomen and temporary abdominal closure techniques in non-trauma patients was recently published [70]. The search identified 74 studies describing 78 patient series, comprising 4,358 patients of which 3,461 (79 %) had peritonitis. The best results in terms of achieving delayed fascial closure and reducing the risk of entero-atmospheric fistula were shown for NPT with continuous fascial traction. Despite that the authors concluded that the overall quality of the available evidence was poor, and uniform recommendations cannot be made.

In 2012 a retrospective analysis evaluating the use of vacuum-assisted closure and mesh-mediated fascial traction (VACM) as temporary abdominal closure was published. The study compared 50 patients treated with VACM and 54 using non-traction techniques (control group). VACM resulted in a higher fascial closure rate and lower planned hernia rate compared with methods that did not provide fascial traction [71].

Recently a prospective study of 108 patients with diffuse peritonitis and open abdomen enrolled from January 2006 to December 2013 was published. Sixty-nine patients were treated with mesh-foil laparostomy without negative pressure and 49 with vacuum-assisted closure (VAC) [72].

The results clearly suggested the advantage of negative pressure therapy in comparison to the temporary abdominal closure with mesh-foil laparostomy in the cases with severe diffuse peritonitis.

While VAC was associated with low incidence of enteroatmospheric fistula, the fascial approximation techniques (mesh mediated fascial traction and dynamic retention sutures) were associated with less lateral fascial retraction and significant increase of fascial closure. Even if there is lack of good quality of evidence, the combination of NPT and fascial approximation technique is the most promising TAC method [65].

### Nutrition support

Nutrition is known to be a key component in the recovery of patients following severe injury or abdominal sepsis. The open abdomen is a significant source of protein and nitrogen loss (up to 2 g per day) [73], failure to account for this may lead to malnutrition with overall poor outcome. Enteral feeding does not increase the risk of ACS [74].

Whilst, there is no strong evidence of enteral nutrition policy in patients with OA, multiple retrospective reviews and one prospective study demonstrate that enteral nutrition is safe within 36 h to 4 days of damage control laparotomy [75–78]. These studies have demonstrated increased rates of fascial closure and demonstrated decreased infectious complications with early enteral nutrition.

### Delayed abdominal wall reconstruction

Primary fascial closure of the abdomen is occasionally not possible. A plan must be made to prevent a ventral hernia development in this scenario.

Delayed repair by bridging biological meshes has been proposed [79]. The role of biological mesh in the management of open abdomen has not been completely clarified and may result in bulging or recurrences [80]. In 2012 the Italian Biological Prosthesis Working Group (IBPWG) proposed a decisional algorithm in using biological meshes to restore abdominal wall defects [81]. Well-controlled prospective investigations are necessary to better define the role of biological meshes.

If definitive fascial closure is not possible another option may be skin only closure to cover the exposed viscera and protect it, minimizing further injury to the exposed bowel. Ostomies should be placed as lateral as possible to be adequate [82].

The open abdomen may result in late development of large, debilitating hernias of the abdominal wall which require delayed repair with synthetic meshes after hospital discharge.

A useful technique for the repair of large midline abdominal wall hernias as consequence of open is abdomen component separation technique (CST).

This technique for reconstructing abdominal wall defects without the use of prosthetic material was described in 1990, by Ramirez et al. [83, 84].

The technique is based on enlargement of the abdominal wall surface by mobilisation of the muscular layers without severing the innervation and blood supply of the muscles [85, 86].

In a prospective randomized trial comparing component separation technique with bridging the defect with prosthetic material, CST was found to be superior to the insertion of prosthetic material. Nevertheless a similar reherniation rate was found after a after a 24 months follow-up [87]. The scars or ostomies in the anterolateral part of abdominal wall can make separation of abdominal layers difficult and can limit the feasibility of CST.

### Enteroatmospheric fistula

The development of enteroatmospheric fistula is the most serious local complication in patients with OA. The exposed bowel is at risk of fistulisation, especially in long standing OA and in the presence of synthetic meshes and residual infection [88].

The formation of an enteroatmospheric fistulas in an OA is a challenge for surgeons. Spontaneous closure is rare, because of they have no well-vascularized overlying tissue and no true fistula tract [89, 90].

Key components of management enteroatmospheric fistulas include adequate delivery of nutrition, electrolyte/fluid deficit correction and adequate broader spectrum antimicrobial therapy. A useful acronym to apply to the management of such patients is “SNAP,” representing management of Sepsis and Skin care, Nutritional support, definition of intestinal Anatomy, and development of a surgical Procedure to deal with the fistula [91].

In literature several methods to manage such fistulas have been described.

The most common strategies for enteroatmospheric fistulas management are:

- control and divert the fistula output, using systems such as nipple or ring with negative pressure therapy applied over tissue around to allow granulation;
- skin grafting over granulation tissue around the fistula, to apply a colostomy bag;
- definitive surgical treatment of fistula when the patient is fully recovered, without sepsis and in good nutritional status, usually after 6 to 12 months.

However, whenever possible or technically feasible, the most effective therapy of enteroatmospheric fistula is prevention and closure of open abdomen as soon as possible.

The open abdomen procedure, although a lifesaving technique, presents a clinical challenge because it may be associated with significant morbidity.

Ideally, the fascia should be closed in patients whose adequate source control is performed with no further planned surgical intervention, severe sepsis is controlled and fascial closure is feasible without relevant increase of IAP.

Fascia should be definitively closed as soon as possible (early facial closure).

Negative pressure wound therapy techniques in combination with fascial approximation techniques should be used for delayed closure.

## Conclusions

OA as part of a damage control strategy may be a life-saving strategy in a well-selected group of surgical patients with severe abdominal sepsis. Once severe sepsis has been controlled, definitive surgical reconstruction should be performed within 48 h.

Rapid closure by negative pressure and dynamic retention sutures of the fascia should be the primary objective in the management of these patients, in order to prevent severe morbidity such as fistulae, loss of domain and massive incisional hernias. The open abdomen strategy presents a clinical challenge that is associated with significant morbidity and OA should be used in the right patients at the right time. Even with the lack of strong evidence in international literature, OA may be an important option in the surgeon’s strategy for the treatment of severe abdominal sepsis. Well-designed prospective and randomised studies are required to adequately define the role of OA and negative pressure in managing patients with abdominal sepsis.

Surgeons should be aware of physiopathology of sepsis and always keep in mind the rationale of open abdomen to be able to use it in the right patient at the correct time. A correct management is crucial to avoid severe complications.

Despite lack of high quality data, OA may be an important option in the surgeon’s armamentarium for the treatment of severe peritonitis.

Well-designed prospective studies are required *to better define the role of OA in managing patients with abdominal sepsis*.

## Appendix

### Appendix 1. OA classification system [19]

1. No fixation
  1. Clean, no fixation
  2. Contaminated, no fixation
  3. Enteric leak, no fixation
2. Developing fixation
  2 A Clean, developing fixation
  2 B Contaminated, developing fixation
  2 C Enteric leak, developing fixation
3. Frozen abdomen
  3 A Clean, frozen abdomen
  3 B Contaminated, frozen abdomen
4. Established enteroatmospheric fistula, frozen abdomen

## References

[1] Gupta S, Kaushik R (2006). Peritonitis - the Eastern experience. World J Emerg Surg..

[2] Sartelli M (2010). A focus on intra-abdominal infections. World J Emerg Surg..

[3] Calandra T, Cohen J (2005). International Sepsis Forum Definition of Infection in the ICU Consensus Conference. The international sepsis forum consensus conference on definitions of infection in the intensive care unit. Crit Care Med.

[4] Mishra SP, Tiwary SK, Mishra M, Gupta SK (2014). An introduction of Tertiary Peritonitis. J Emerg Trauma Shock.

[5] Sartelli M, Catena F, Di Saverio S, Ansaloni L, Malangoni M, Moore EE (2014). Current concept of abdominal sepsis: WSES position paper. World J Emerg Surg.

[6] LaRosa SP (2002). Sepsis: Menu of new approaches replaces one therapy for all. Cleve Clin J Med.

[7] Levy MM, Fink MP, Marshall JC, Abraham E, Angus D, Cook D (2001). SCCM/ ESICM/ACCP/ATS/ SIS International Sepsis definitions conference. Crit Care Med.

[8] Dellinger RP, Levy MM, Carlet JM, Bion J, Parker MM, Jaeschke R (2008). Surviving sepsis campaign: international guidelines for management of severe sepsis and septic shock: 2008. Crit Care Med.

[9] Bone RC, Balk RA, Cerra FB, Dellinger RP, Fein AM, Knaus WA (1992). American College of Chest Physicians/Society of Critical Care Medicine Consensus Conference: definitions for sepsis and organ failure and guidlines for the use of innovative therapies in sepsis. Chest.

[10] Esteban A, Frutos-Vivar F, Ferguson ND, Peñuelas O, Lorente JA, Gordo F (2007). Sepsis incidence and outcome: contrasting the intensive care unit with the hospital ward. Crit Care Med.

[11] Angus DC, van der Poll T (2013). Severe sepsis and septic shock. N Engl J Med.

[12] Sartelli M, Catena F, Ansaloni L, Coccolini F, Corbella D, Moore EE (2014). Complicated intra-abdominal infections worldwide: the definitive data of the CIAOW Study. World J Emerg Surg..

[13] Koperna T, Semmler D, Marian F (2001). Risk stratification in emergency surgical patients: is the APACHE II score a reliable marker of physiological impairment?. Arch Surg.

[14] Kiewiet JJ, van Ruler O, Boermeester MA, Reitsma JB (2013). A decision rule to aid selection of patients with abdominal sepsis requiring a relaparotomy. BMC Surg..

[15] Van Ruler O, Mahler CW, Boer KR, Reuland EA, Gooszen HG, Opmeer BC (2007). Comparison of on-demand vs planned relaparotomy strategy in patients with severe peritonitis: a randomized trial. JAMA.

[16] Leppäniemi AK (2010). Laparostomy: why and when?. Crit Care.

[17] Ivatury RR (2009). Update on open abdomen management: achievements and challenges. World J Surg.

[18] Robledo FA, Luque-de-León E, Suárez R, Sánchez P, de-la-Fuente M, Vargas A (2007). Open versus closed management of the abdomen in the surgical treatment of severe secondary peritonitis: a randomized clinical trial. Surg Infect (Larchmt).

[19] Kirkpatrick AW, Roberts DJ, De Waele J, Jaeschke R, Malbrain ML, De Keulenaer B (2013). Pediatric Guidelines Sub-Committee for the World Society of the Abdominal Compartment Syndrome. Intra-abdominal hypertension and the abdominal compartment syndrome: updated consensus definitions and clinical practice guidelines from the World Society of the Abdominal Compartment Syndrome. Intensive Care Med.

[20] Schein M (2002). Surgical management of intra-abdominal infection: is there any evidence?. Langenbecks Arch Surg.

[21] De Siqueira J, Tawfiq O, Garner J (2014). Managing the open abdomen in a district general hospital. Ann R Coll Surg Engl.

[22] Yuan Y, Ren J, He Y (2013). Current status of the open abdomen treatment for intra-abdominal infection. Gastroenterol Res Pract.

[23] Bjorck M, Bruhin A, Cheatham M (2009). Classification – important step to improve management of patients with an open abdomen. World J. Surg..

[24] Marshall JC, al Naqbi A (2009). Principles of source control in the management of sepsis. Crit Care Clin.

[25] Stone HH, Strom PR, Mullins RJ (1983). Management of the major coagulopathy with onset during laparotomy. Ann Surg.

[26] Burch JM, Ortiz VB, Richardson RJ, Martin RR, Mattox KL, Jordan GL (1992). Abbreviated laparotomy and planned reoperation for critically injured patients. Ann Surg.

[27] Moore LJ, Moore FA (2012). Epidemiology of sepsis in surgical patients. Surg Clin North Am.

[28] van Ruler O, Lamme B, Gouma DJ, Reitsma JB, Boermeester MA (2007). Variables associated with positive findings at relaparotomy in patients with secondary peritonitis. Crit Care Med.

[29] Koperna T, Schulz F (2000). Relaparotomy in peritonitis: prognosis and treatment of patients with persisting intraabdominal infection. World J Surg.

[30] Lamme B, Mahler CW, van Ruler O, Gouma DJ, Reitsma JB, Boermeester MA (2006). Clinical predictors of ongoing infection in secondary peritonitis: systematic review. World J Surg.

[31] Hinsdale JG, Jaffe BM (1984). Re-operation for intra-abdominal sepsis. Indications and results in modern critical care setting. Ann Surg.

[32] van Ruler O, Kiewiet JJ, Boer KR, Lamme B, Gouma DJ, Boermeester MA (2011). Failure of available scoring systems to predict ongoing infection in patients with abdominal sepsis after their initial emergency laparotomy. BMC Surg..

[33] van Ruler O, Lamme B, de Vos R, Obertop H, Reitsma JB, Boermeester MA (2008). Decision making for relaparotomy in secondary peritonitis. Dig Surg.

[34] Bailey J, Shapiro MJ (2000). Abdominal compartment syndrome. Crit Care.

[35] Van Hee R (2007). Historical highlights in concept and treatment of abdominal compartment syndrome. Acta Clin Belg Suppl.

[36] Demetriades D (2012). Total management of the open abdomen. Int Wound J.

[37] Papavramidis TS, Marinis AD, Pliakos I, Kesisoglou I, Papavramidou N (2011). Abdominal compartment syndrome - Intra-abdominal hypertension: Defining, diagnosing, and managing. J Emerg Trauma Shock.

[38] Ordoñez CA, Pino LF, Badiel M, Sánchez AI, Loaiza J, Ballestas L (2011). Safety of performing a delayed anastomosis during damage control laparotomy in patients with destructive colon injuries. J Trauma.

[39] Morris JA, Eddy VA, Blinman TA, Rutherford EJ, Sharp KW (1993). The staged celiotomy for trauma. Issues in unpacking and reconstruction. Ann Surg.

[40] Ordonez CA, Sanchez AI, Pineda JA (2010). Deferred primary anastomosis versus diversion in patients with severe secondary peritonitis managed with staged laparotomies. World J Surg.

[41] Kafka-Ritsch R, Birkfellner F, Perathoner A, Raab H, Nehoda H, Pratschke J (2012). Damage control surgery with abdominal vacuum and delayed bowel reconstruction in patients with perforated diverticulitis Hinchey III/IV. J Gastroenterol Surg.

[42] Sartelli M, Viale P, Catena F, Ansaloni L, Moore E, Malangoni M (2013). 2013 WSES guidelines for management of intra-abdominal infections. World J Emerg Surg..

[43] Leroy G, Lambiotte F, Thévenin D, Lemaire C, Parmentier E, Devos P (2011). Evaluation of "Candida score" in critically ill patients: a prospective, multicenter, observational, cohort study. Ann Intensive Care.

[44] Eggimann P, Que YA, Revelly JP, Pagani JL (2015). Preventing invasive candida infections. Where could we do better?. J Hosp Infect.

[45] Demetriades D, Salim A (2014). Management of the open abdomen. Surg Clin North Am.

[46] Regner JL, Kobayashi L, Coimbra R (2012). Surgical strategies for management of the open abdomen. World J Surg.

[47] Godat L, Kobayashi L, Costantini T, Coimbra R (2013). Abdominal damage control surgery and reconstruction: World society of emergency surgery position paper. World J Emerg Surg.

[48] Chen Y, Ye J, Song W, Chen J, Yuan Y, Ren J (2014). Comparison of outcomes between early fascial closure and delayed abdominal closure in patients with open abdomen: a systematic review and meta-analysis. Gastroenterol Res Pract..

[49] Paul JS, Ridolfi TJ (2012). A case study in intra-abdominal sepsis. Surg Clin North Am.

[50] Lambertz A, Mihatsch C, Röth A, Kalverkamp S, Eickhoff R, Neumann UP (2015). Fascial closure after open abdomen: Initial indication and early revisions are decisive factors - A etrospective cohort study. Int J Surg..

[51] Padalino P, Dionigi G, Minoja G, Carcano G, Rovera F, Boni L (2010). Fascia-to-fascia closure with abdominal topical negative pressure for severe abdominal infections: preliminary results in a department of general surgery and intensive care unit. Surg Infect (Larchmt).

[52] Regli A, De Keulenaer B, De Laet I, Roberts D, Dabrowski W, Malbrain ML (2015). Fluid therapy and perfusional considerations during resuscitation in critically ill patients with intra-abdominal hypertension. Anaesthesiol Intensive Ther.

[53] Teixeira PG, Salim A, Inaba K, Brown C, Browder T, Margulies D (2008). A prospective look at the current state of open abdomens. Am Surg.

[54] Webb LH, Patel MB, Dortch MJ, Miller RS, Gunter OL, Collier BR (2012). Use of a furosemide drip does not improve earlier primary fascial closure in the open abdomen. J Emerg Trauma Shock.

[55] Schein M, Saadia R, Jamieson JR, Decker GA (1986). The 'sandwich technique' in the management of the open abdomen. Br J Surg.

[56] Kreis BE, de Mol van Otterloo AJ, Kreis RW (2013). Open abdomen management: a review of its history and a proposed management algorithm. Med Sci Monit..

[57] Brock WB, Barker DE, Burns RP (1995). Temporary closure of open abdominal wounds: the vacuum pack. Am Surg.

[58] Campbell A, Chang M, Fabian T, Franz M, Kaplan M, Moore F (2009). Management of the open abdomen: from initial operation to definitive closure. Am Surg.

[59] Barker DE, Green JM, Maxwell RA, Smith PW, Mejia VA, Dart BW (2007). Experience with vacuum-pack temporary abdominal wound closure in 258 trauma and general and vascular surgical patients. J Am Coll Surg.

[60] Plaudis H, Rudzats A, Melberga L, Kazaka I, Suba O, Pupelis G (2012). Abdominal negative-pressure therapy: a new method in countering abdominal compartment and peritonitis - prospective study and critical review of literature. Ann Intensive Care.

[61] Kubiak BD, Albert SP, Gatto LA, Snyder KP, Maier KG, Vieau CJ (2010). Peritoneal negative pressure therapy prevents multiple organ injury in a chronic porcine sepsis and ischemia/reperfusion model. Shock.

[62] Rausei S, Amico F, Frattini F, Rovera F, Boni L, Dionigi G (2014). A review on vacuum-assisted closure therapy for septic peritonitis open abdomen management. Surg Technol Int..

[63] Boele van Hensbroek P, Wind J, Dijkgraaf MG, Busch OR, Goslings JC (2009). Temporary closure of the open abdomen: a systematic review on delayed primary fascial closure in patients with an open abdomen. World J Surg.

[64] Verdam FJ, Dolmans DE, Loos MJ, Raber MH, de Wit RJ, Charbon JA (2011). Delayed primary closure of the septic open abdomen with a dynamic closure system. World J Surg.

[65] Terzi C, Egeli T, Canda AE, Arslan NC (2014). Management of enteroatmospheric fistulae. Int Wound J..

[66] Di Saverio S, Villani S, Biscardi A, Giorgini E, Tugnoli G (2011). Open abdomen with concomitant enteroatmospheric fistula: validation, refinements, and adjuncts to a novel approach. J Trauma..

[67] Di Saverio S, Tarasconi A, Inaba K, Navsaria P, Coccolini F, Costa Navarro D (2015). Open abdomen with concomitant enteroatmospheric fistula: attempt to rationalize the approach to a surgical nightmare and proposal of a clinical algorithm. J Am Coll Surg.

[68] Fortelny RH, Hofmann A, Gruber-Blum S, Petter-Puchner AH, Glaser KS (2014). Delayed closure of open abdomen in septic patients is facilitated by combined negative pressure wound therapy and dynamic fascial suture. Surg Endosc.

[69] Ferreira F, Barbosa E, Guerreiro E, Fraga GP, Nascimento B, Rizoli S (2013). Sequential closure of the abdominal wall with continuous fascia traction (using mesh or suture) and negative pressure therapy. Rev Col Bras Cir.

[70] Atema JJ, Gans SL, Boermeester MA (2015). Systematic review and meta-analysis of the open abdomen and temporary abdominal closure techniques in non-trauma patients. World J Surg..

[71] Rasilainen SK, Mentula PJ, Leppäniemi AK (2012). Vacuum and mesh-mediated fascial traction for primary closure of the open abdomen in critically ill surgical patients. Br J Surg.

[72] Mutafchiyski VM, Popivanov GI, Kjossev KT, Chipeva S. Open abdomen and VAC® in severe diffuse peritonitis. J R Army Med Corps. 2015 Feb 23. doi: 10.1136/jramc-2014-000386. [Epub ahead of print]. 10.1136/jramc-2014-000386 25712560

[73] Cheatham ML, Safcsak K, Brzezinski SJ, Lube MW (2007). Nitrogen balance, protein loss, and the open abdomen. Crit Care Med.

[74] Cothren CC, Moore EE, Ciesla DJ, Johnson JL, Moore JB, Haenel JB (2004). Postinjury abdominal compartment syndrome does not preclude early enteral feeding after definitive closure. Am J Surg.

[75] Collier B, Guillamondegui O, Cotton B, Donahue R, Conrad A, Groh K (2007). Feeding the open abdomen. JPEN J Parenter Enteral Nutr.

[76] Burlew CC, Moore EE, Cuschieri J, Jurkovich GJ, Codner P, Nirula R (2012). Who should we feed? Western trauma association multi-institutional study of enteral nutrition in the open abdomen after injury. Trauma Acute Care Surg.

[77] Byrnes MC, Reicks P, Irwin E (2010). Early enteral nutrition can be successfully implemented in trauma patients with an “open abdomen”. Am J Surg.

[78] Dissanaike S, Pham T, Shalhub S, Warner K, Hennessy L, Moore EE (2008). Effect of immediate enteral feeding on trauma patients with an open abdomen: protection from nosocomial infections. J Am Coll Surg.

[79] Kissane NA, Itani KM (2012). A decade of ventral incisional hernia repairs with biologic acellular dermal matrix: what have we learned?. Plast Reconstr Surg.

[80] de Moya MA, Dunham M, Inaba K (2008). Long-term outcome of acellular dermal matrix when used for large traumatic open abdomen. J Trauma..

[81] Coccolini F, Agresta F, Bassi A, Catena F, Crovella F, Ferrara R (2012). Proposal for a decisional model in using biological prosthesis. World J Emerg Surg.

[82] Jernigan TW, Fabian TC, Croce MA, Moore N, Pritchard FE, Minard G (2003). Staged management of giant abdominal wall defects: acute and long-term results. Ann Surg.

[83] Cheesborough JE, Park E, Souza JM, Dumanian GA (2014). Staged management of the open abdomen and enteroatmospheric fistulae using split-thickness skin grafts. Am J Surg.

[84] Ramirez OM, Ruas E, Lee Dellon A (1990). “Components separation” method for closure of abdominal wall defects: an anatomic and clinical study. Plast Reconstr Surg.

[85] De Vries Reilingh TS, van Goor H, Rosman C, Bemelmans MH, de Jong D, van Nieuwenhoven EJ (2003). “Components separation technique” for the repair of large abdominal wall hernias. J Am Coll Surg.

[86] Rasilainen SK, Mentula PJ, Leppäniemi AK. Components separation technique is feasible for assisting delayed primary fascial closure of open abdomen. Scand J Surg. 2015;13. 10.1177/1457496915586651 25972489

[87] DiBello JN, Moore JH (1996). Sliding myofascial flap of the rectus abdominis muscle for the closure of recurrent ventral hernias. Plast Reconstr Surg.

[88] de Vries Reilingh TS, van Goor H, Charbon JA, Rosman C, Hesselink EJ, van der Wilt GJ (2007). Repair of giant midline abdominal wall hernias: “components separation technique” versus prosthetic repair: interim analysis of a andomized controlled trial. World J Surg.

[89] Losanoff JE, Richman BW, Jones JW (2003). Intestinal fistulization in the open treatment of peritonitis. Am J Surg.

[90] Mastboom WJ, Kuypers HH, Schoots FJ, Wobbes T (1989). Small-bowel perforation complicating the open treatment of generalized peritonitis. Arch Surg.

[91] Kaushal M, Carlson GL (2004). Management of enterocutaneous fistulas. Clin Colon Rectal Surg.

